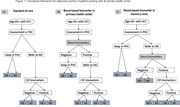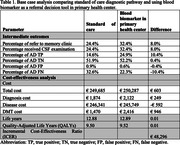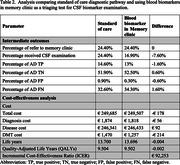# Blood biomarkers for Alzheimer’s disease diagnosis in the context of disease‐modifying treatment: a cost‐utility analysis

**DOI:** 10.1002/alz.086085

**Published:** 2025-01-09

**Authors:** Sandar Aye, Ron Handels, Bengt Winblad, Linus Jönsson

**Affiliations:** ^1^ Karolinska Institutet, Stockholm, Stockholm Sweden; ^2^ Maastricht University; Department of Psychiatry and Neuropsychology; Alzheimer Centre Limburg; School for Mental Health and Neurosciences, Maastricht Netherlands; ^3^ Div of neurogeriatrics, Dept for Neurobiology, Care Sciences and Society, Karolinska Institutet, Solna Sweden; ^4^ Theme Inflammation and Aging, Karolinska University Hospital, Stockholm Sweden; ^5^ Division of Neurogeriatrics, Karolinska Institutet, Stockholm Sweden; ^6^ Karolinska Institutet, Solna, Stockholm Sweden

## Abstract

**Background:**

Recent developments in blood biomarkers (BBM) have shown promising results in diagnosing amyloid pathology in Alzheimer’s Disease (AD). However, information on how these BBMs can best be used in clinical settings to optimise clinical decision‐making and long‐term health outcomes for individuals with AD is still lacking. We aim to assess the potential clinical and economic value of BBM in AD diagnosis within the context of disease‐modifying treatment (DMT).

**Method:**

We developed a decision analytic model to evaluate the long‐term health outcomes of using BBM in AD diagnosis. We compared standard of care (SOC) diagnosis workflow to the integration of BBM as a (1) referral decision tool in primary health center (PHC) and (2) triaging tool for invasive CSF examination in specialist memory clinic (MC). The conceptual framework is illustrated in Figure 1. We combined a decision tree and a Markov model to simulate the patient’s diagnostic journey, treatment decisions following diagnosis and long‐term health outcomes. We applied a cost‐effective price of DMT identified from price threshold analysis. A cost‐utility analysis was conducted from the societal perspective using a one‐year cycle length and a 30‐year time horizon. The outcomes were reported in percentage of correct diagnosis, costs (in 2022 Euros), quality‐adjusted life year (QALY), and incremental cost‐effectiveness ratios (ICER).

**Result:**

Compared to SOC, integrating BBM in PHC increased patient referrals by 8% and true positive AD diagnoses by 10.4%. The lifetime costs for individuals diagnosed with AD were € 249,685 and €250,287, and QALYs were 9.5 and 9.52 in SOC and PHC pathways, respectively. The cost increments were €603, and QALYs gained were 0.01, resulting in an ICER of €48,296. Using BBM in MC reduced the exposure to invasive CSF procedures and costs but also reduced true positive AD diagnoses and QALYs. Detailed results were presented in Tables 1 & 2.

**Conclusion:**

Using BBM at PHC to make referral decisions might increase initial diagnostic costs but can prevent high costs associated with disease progression, providing a cost‐effective DMT is available, whereas using BBM in MC could reduce the initial evaluation cost but incur high costs associated with disease progression.